# Experimental Array for Generating Dual Circularly-Polarized Dual-Mode OAM Radio Beams

**DOI:** 10.1038/srep40099

**Published:** 2017-01-10

**Authors:** Xu-Dong Bai, Xian-Ling Liang, Yun-Tao Sun, Peng-Cheng Hu, Yu Yao, Kun Wang, Jun-Ping Geng, Rong-Hong Jin

**Affiliations:** 1 Department of Electronics Engineering, Shanghai Jiao Tong University, Shanghai 200240, China; 2 China Academy of Aerospace Electronics Technology, Shanghai Aerospace Electronics Co., Ltd, Shanghai 201821, China

## Abstract

Recently, vortex beam carrying orbital angular momentum (OAM) for radio communications has attracted much attention for its potential of transmitting multiple signals simultaneously at the same frequency, which can be used to increase the channel capacity. However, most of the methods for getting multi-mode OAM radio beams are of complicated structure and very high cost. This paper provides an effective solution of generating dual circularly-polarized (CP) dual-mode OAM beams. The antenna consists of four dual-CP elements which are sequentially rotated 90 degrees in the clockwise direction. Different from all previous published research relating to OAM generation by phased arrays, the four elements are fed with the same phase for both left-hand circular polarization (LHCP) and right-hand circular polarization (RHCP). The dual-mode operation for OAM is achieved through the opposite phase differences generated for LHCP and RHCP, when the dual-CP elements are sequentially rotated in the clockwise direction. The measured results coincide well with the simulated ones, which verified the effectiveness of the proposed design.

Nowadays, in order to supply more capacity and support various wide-band services, it is a compulsory trend to increase the spectral resources within the context of wireless communications. The technological evolution in telecommunications has led to the development of many techniques showing the ability for increasing the spectral efficiency, such as polarization-division multiplexing (PDM) and orthogonal frequency-division multiplexing (OFDM)^1^. In addition to the above-mentioned methods, it has been recently proposed a new approach based on the use of radio waves carrying orbital angular momentum (OAM). Since each of the OAM eigenstates with integral topological charges is orthogonal and independent of the other, it shows potential of transmitting multiple signals simultaneously at the same frequency with low inter-mode crosstalk in future short range communications, by creating multiple sub-channels of propagation corresponding to the twisting degree of the electromagnetic wave^2^.

Under the rule of wave-particle duality, it was proven that electromagnetic wave carries both linear momentum and angular momentum, and the total angular momentum is composed of spin angular momentum (SAM), which refers to the polarization state, and orbital angular momentum (OAM), which is related to the spatial distributions of the field magnitude and phase^3^. The SAM describing the polarization state is well known and widely exploited in operating communication system starting around 1935, when its mechanical properties was demonstrated both theoretically and experimentally by Beth^4^; however, OAM was not fascinating until Allen *et al*. investigated in 1992 that a beam comprising 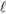 helical phase fronts, described by a phase term 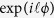, carries an OAM of 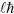 per photon, where 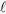 can take any integer value^5^. At first, the utilizations of OAM were mainly concentrated on the optical regime, and the capacity of optical communication systems was largely enhanced by introducing OAM^6^,^7^. Recently, radio beams were also prepared in pure OAM states and superpositions, which proved the prospect for OAM application in the low frequency radio domain^8^. Afterwards, the dielectric or FSS spiral phase plate^9^,^10^,^11^, spiral reflector^12^, twisted parabolic reflector^13^,^14^,^15^, the plane reflectarray^16^, and circular arrays with the shunt-fed or series-fed networks^17^,^18^,^19^ were introduced to generate a single-mode electromagnetic wave carrying non-zero OAM. To get dual-mode OAM beams, a stacked antenna composed of two circular arrays was proposed to achieve a dual-channel OAM wireless communication^20^, but its construction is relative complicated with two dielectric boards. For the multi-mode OAM operation, the circular array was fed with variable phases by phased array system^21^,^22^, the Butler matrix^23^ or Rotman lens^24^ to generate OAM waves with different modes, but their design and architecture are all very complicated, which restrict the application.

In this paper, we present a succinct solution of generating dual circularly-polarized (CP) dual-mode OAM radio beams. By sequentially rotating the dual-CP elements in the clockwise direction, the opposite phase differences are generated for left-handed CP (LHCP) and right-handed CP (RHCP), thus the dual-mode operation for OAM is achieved. Since the four elements are fed with the same phase for both LHCP and RHCP, the feed networks of the array are much simpler and ease of implementation. Simulation results and experimental measurements validate the effectiveness of the proposed design for generating dual-mode OAM beams.

## Results

### Theory and design for the dual OAM modes antenna

**Figure 1:**
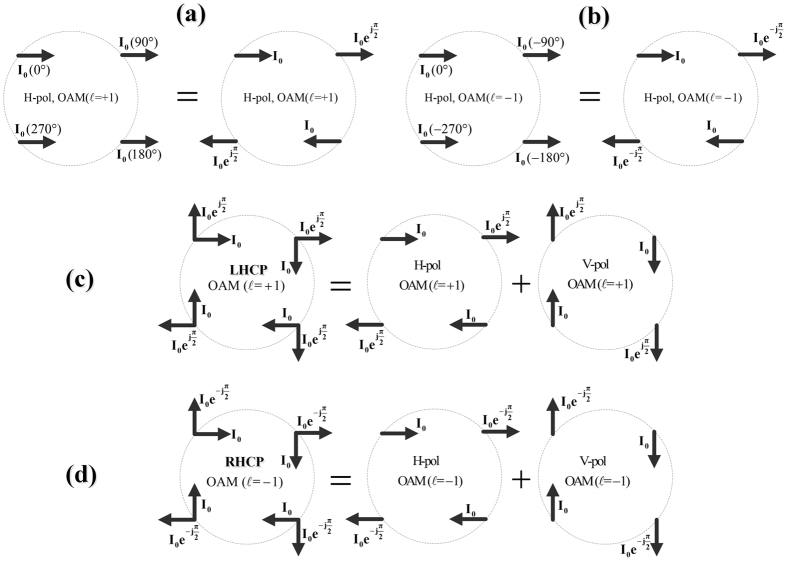
Configuration of a typical four-element OAM-generating array. (**a**) Horizontally polarized, OAM mode 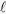 = +1. (**b**) Horizontally polarized, OAM mode 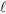 = −1. (**c**) The LHCP antenna array and its polarization decomposition. (**d**) The RHCP antenna array and its polarization decomposition.

For a typical *N*-element OAM-generating circular phased array, the element should be fed with the uniform amplitude but with a successive phase difference for the successive elements, which is 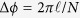 (where 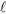 is the topological charge, and 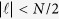)^8^,^21^. Figure 1(a,b) gives the geometry of a typical 4-element dipole array for OAM beams with modes 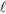 = ±1. The phase difference for the successive elements in clockwise direction is 90° for OAM mode 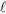 = +1, while the phase difference for OAM mode 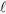 = −1 is −90°.

In our work, four dual-CP patch elements are employed for building the OAM-generating array. Different from the common methods of array arrangement shown in Fig. 1(a,b), the four elements are fed with the same phase, but are sequentially rotated 90 degrees in the clockwise direction.

The configuration of a LHCP array using sequentially rotated elements and its polarization decomposition are shown in Fig. 1(c), since a circular polarization can be seen as the superposition of two orthogonal line polarizations with 90° phase difference. When comparing Fig. 1(c) with Fig. 1(a), we can easily find that a 90° phase difference is obtained in successive elements for both horizontal and vertical polarizations after sequentially rotating the LHCP elements with 90° in the clockwise direction, thus the OAM mode 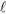 = +1 is generated. As for a RHCP array shown in Fig. 1(d), when compared with Fig. 1(b), we can see that a −90° phase difference is obtained in successive elements for both horizontal and vertical polarizations after sequentially rotating the RHCP elements with 90° in the clockwise direction, thus the OAM mode 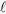 = −1 is generated.

### Structure of the dual OAM modes antenna

**Figure 2:**
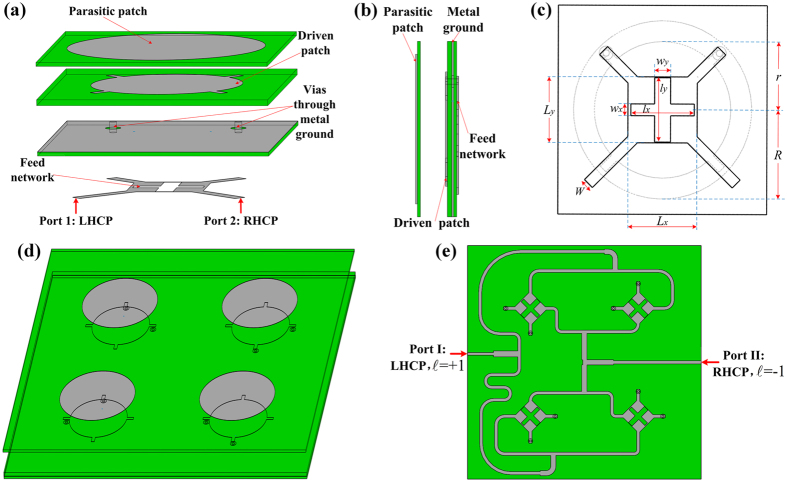
Geometry of the antenna structure. (**a**) Element layered view. (**b**) Element side view. (**c**) Element Upward 2-D view. (**d**) Array side view. (**e**) Array upward 2-D view.

Based on the above-mentioned theories, we use four dual-CP elements to build the OAM-generating array. The four elements are sequentially rotated 90 degrees in the clockwise direction and fed with the same phase. To obtain a wideband operation, a dual-patch element is used, which are composed of three substrate layers and an air-gap, as shown in Fig. 2(a–c). The substrate used for the first and third layers is with the dielectric constant of 3.0 and height of 0.508 mm, while that for the second layer is with the dielectric constant of 3.0 and height of 0.762 mm. The circular parasitic patch is on the top surface of the first layer, with the radius of *R* = 12.8 mm. The driven patch is located on the top surface of second layer, with the radius of *r* = 9.8 mm. Between the first and second layers is an air-gap with a thickness of *G* = 4.335 mm, which is also helpful to obtain the broad bandwidth^25^. The metal ground plane is placed on the top surface of the third layer and fabricated with two apertures for the vias connection between the driven patch and the compact-size semi-lumped hybrid 90°coupler^26^ on the bottom surface of the third layer. The central angle of the two feeding point is 90° to excite the dual-CP operation, and this element structure is very similar to that shown in literature^27^; when one port is excited and the other terminated with a 50-Ω load, the LHCP or RHCP radiation will be generated correspondingly. The parameters of the hybrid coupler are set as follows:*W* = 1.4 mm, *L*_*x*_ = 10.8 mm, *L*_y_ = 10.3 mm, *l*_*x*_ = 10.02 mm, *l*_*y*_ = 10.1 mm, *w*_*x*_ = 1.7 mm, *w*_*y*_ = 2.3 mm.

**Figure 3:**
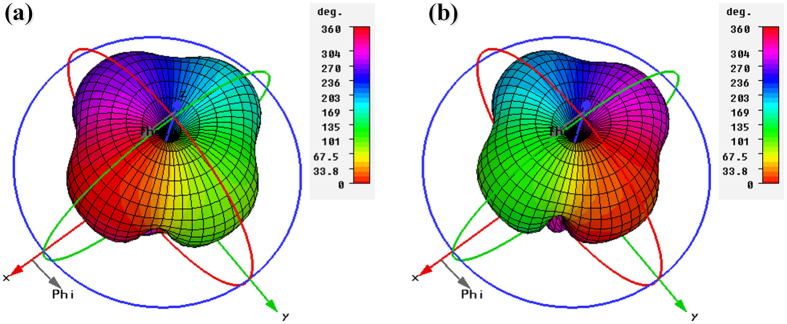
The simulated far-field phase distribution at 4.8 GHz. (**a**) 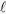 = −1. (**b**) 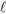 = +1.

Figure 2(d,e) shows the overall geometry of the proposed antenna array, whose overall dimension is 104 mm × 104 mm, and the element spacing is 48 mm. When port I is excited and port II terminated with a 50-Ω load, the antenna array generates the LHCP wave with OAM mode 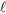 = +1, whereas the RHCP wave with OAM mode 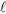 = −1 is generated when port II is excited. The simulated far-field phase distribution of the antenna array is shown in Fig. 3, which is simulated with CST Microwave Studio software based on the finite integration technique (FIT) method. It is observed that, the phase distribution of the OAM waves undergoes a linear variation along the angular coordinate and the changing trends for 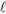 = +1 and 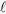 = −1 are in opposite directions.

### Measurement of the antenna

**Figure 4:**
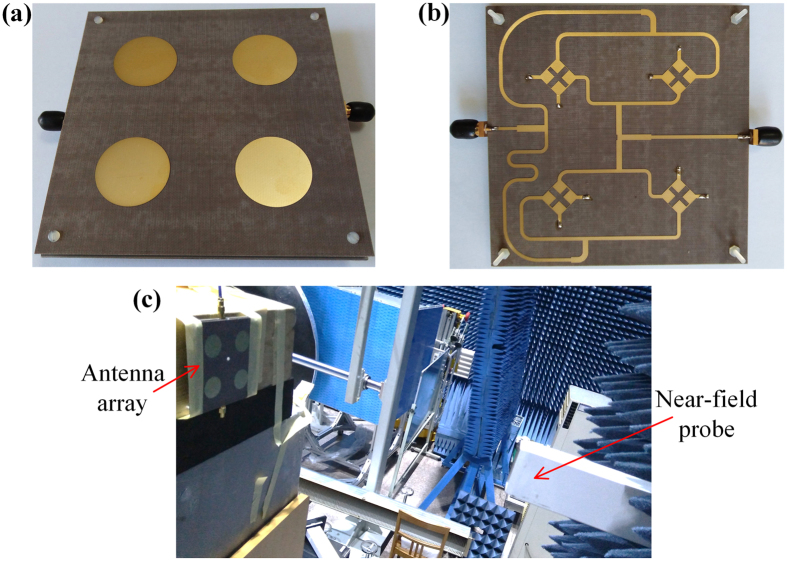
Fabricated prototype of the antenna array. (**a**) Front view. (**b**) Back view. (**c**) The near-field test scenario in the anechoic chamber.

To verify the proposed dual-CP dual-mode OAM array, the prototype lens-fed array was fabricated by using the low-cost commercial printed-circuit-board (PCB) technology, and its measurement is carried out in the anechoic chamber, as shown in Fig. 4.

**Figure 5:**
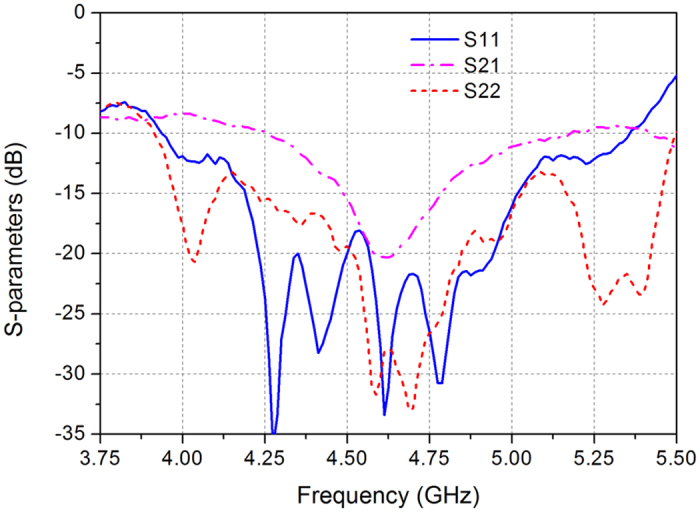
Measured *S*-parameters of the proposed antenna array.

Figure 5 shows the measured *S*-parameters, the measured reflection coefficient |*S*_11_| value for port I is below −10 dB from 3.94 to 5.35 GHz and achieves an impedance bandwidth of over 30%, the measured reflection coefficient |*S*_22_| value for port II is below −10 dB from 3.92 to 5.5 GHz and achieves an impedance bandwidth of 33.5%, and the ports transmission coefficient |*S*_21_| is lower than −10 dB from 4.26 to 5.18 GHz.

**Figure 6:**
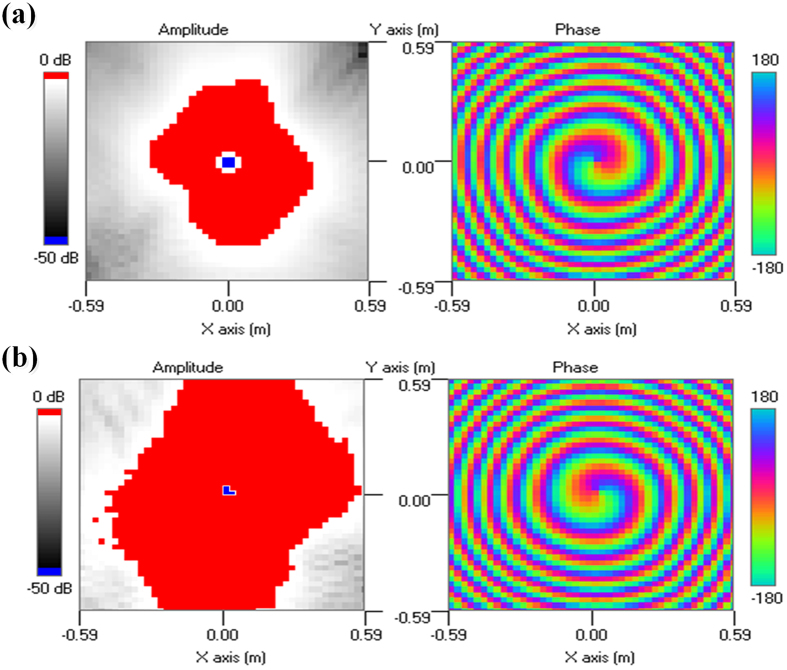
Screenshots of the measured near-field results at 4.80 GHz. (**a**) Field amplitude and phase distribution for 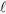 = −1. (**b**) Field amplitude and phase distribution for 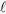 = +1.

The near field distribution of the antenna array is measured with a 3D platform in the near-field anechoic chamber, as shown in Fig. 4(c). An open-ended rectangular waveguide is used as the near-field probe, and the measuring plane is 300 *mm* far from the array plane with a scan range of 1180 *mm *× 1180 *mm*. Figure 6 gives the screenshots of the measured near-field results for both amplitude and phase distributions, and the red shadow in the amplitude distribution represents the high intensity, while the blue shadow in the center of the high-intensity region represents the low intensity. Note that, the on-axis singular-phase null regions are completely revealed, and the ocular null-region sizes of OAM modes 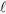 = ±1 seem different but are actually with the same size, and this is mainly due to the large scale range in the default setting of the test instrument. The characteristic vortex phase fronts indicate that the OAM radio beams are created, and the change in color from green to red, blue, and back to green again corresponds to a change in phase of 2*π*. When comparing the positive and negative modes, the phase distribution of 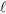 = +1 has the clockwise increase, while that of 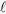 = −1 would have the anticlockwise increase.

**Figure 7:**
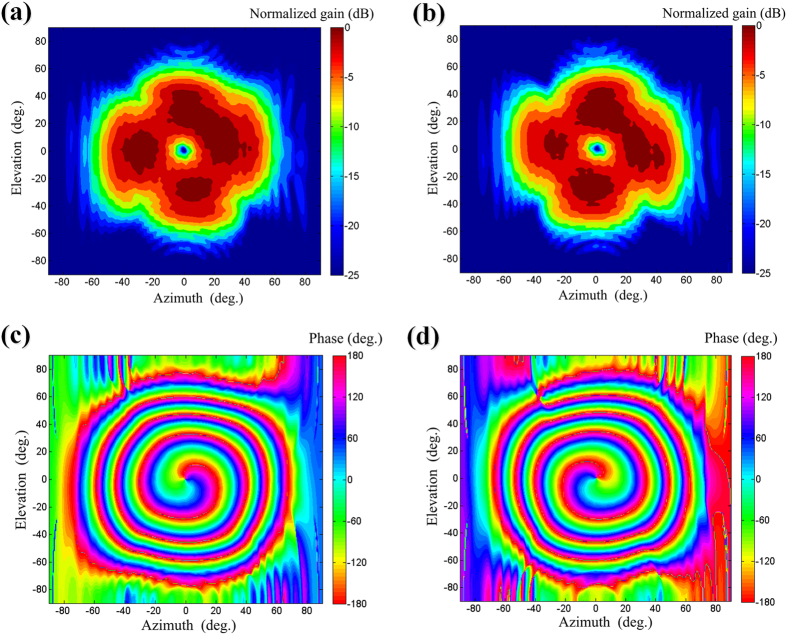
Measured far-field results at 4.8 GHz. Intensity patterns: (**a**) 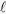 = −1. (**b**) 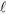 = +1. Phase distribution: (**c**) 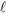 = −1. (d) 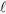 = +1.

The far-field radiations of the array are shown in Fig. 7, which are constructed from the near-field measurement by a two-dimensional fast Fourier transform (FFT) algorithm^28^. Figure 7(a,b) shows the intensity patterns, the doughnut-shaped high-intensity profile is clearly seen, and the null regions of OAM modes 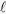 = ±1 have almost the same size. The far-field phase distributions for OAM modes 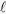 = ±1 are plotted in Fig. 7(c,d), which show the rotating phase distribution.

**Figure 8:**
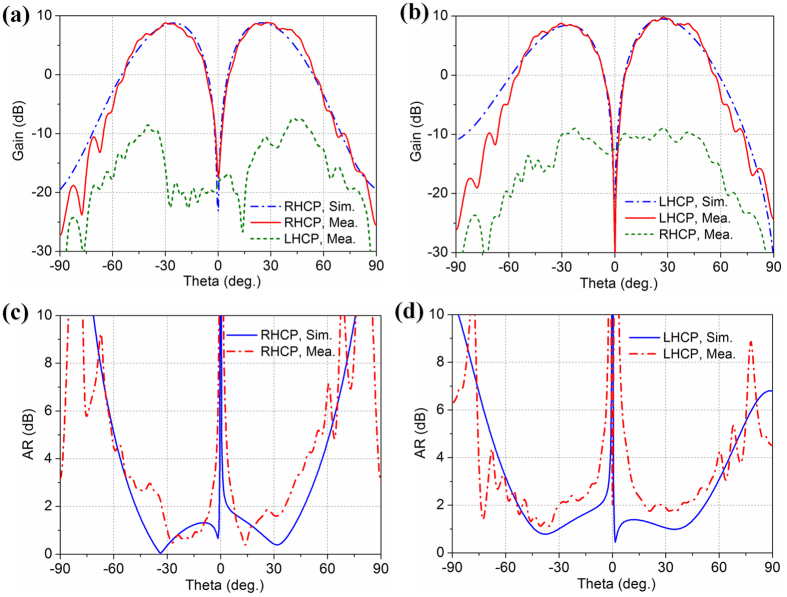
Measured and simulated 2D far-field results at 4.80 GHz. Radiation patterns: (**a**) 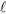 = −1. (**b**) 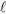 = +1. AR: (**c**) RHCP. (**d**) LHCP.

The simulated and measured 2D far-field radiation patterns are shown in Fig. 8(a,b), which are consistent very well each other. Good dual-CP conical patterns are generated, and the main lobe direction is around 27.5° from the boresight axis, with a 3-dB beamwidth of about 32.5°. The measured array gains are about 9 dBic at 4.80 GHz, and the on-axis null region is 25 dB lower than the high-intensity doughnut-shaped region for both LHCP and RHCP. The intensity of the cross-polarization field is much lower than that of the co-polarization field, and the cross polarization levels are lower than −20 dB for both LHCP and RHCP.

The measured and simulated AR patterns for both LHCP and RHCP are shown in Fig. 8(c,d), and characteristic on-axis phase singularities of the OAM are also revealed on the AR-plots. Although there are some discrepancies due to the manual assembly error, the measured AR-plots are consistent with the simulated ones substantially and are below 2 dB for the peak radiation direction.

## Discussion

A new convenience method of generating dual-CP dual-mode OAM radio beams has been presented in this paper. The dual-mode operation for OAM is achieved through the opposite phase differences generated for LHCP and RHCP, when the dual-CP elements are sequentially rotated in the clockwise direction. Since the four elements are fed with the same phase, the feed networks of the array are much simpler and ease of implementation. The measured results coincide well with the simulated ones, which verify the effectiveness of the proposed design and confirm the generation of the twisted radio beams operating at OAM mode 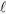 = ±1. Since each of the OAM eigenstates with integral topological charges is orthogonal and independent of the other^2^,^22^, by combining the polarization discrimination with the orthogonal OAM states, the channel isolation of the communication system would have further improvement. All these features make the proposed antenna a promising candidate for radio communications and radar applications.

## Methods

The dual-CP dual-mode OAM radio beams are generated through sequentially rotating the dual-CP elements in the clockwise direction. The dual-CP dual-mode OAM antenna is designed by CST Microwave Studio and manufactured on the Arlon AD300 substrates using the PCB technology.

The measurement is carried out with a 3D platform in the anechoic chamber based on the Agilent Vector Network Analyzer 8722ES, and both intensity and phase distributions of the dual-mode OAM waves can be measured.

## Additional Information

**How to cite this article**: Bai, X.-D. *et al*. Experimental Array for Generating Dual Circularly-Polarized Dual-Mode OAM Radio Beams. *Sci. Rep.* **7**, 40099; doi: 10.1038/srep40099 (2017).

**Publisher's note:** Springer Nature remains neutral with regard to jurisdictional claims in published maps and institutional affiliations.
